# Exploring the Addition of Herbal Residues on Fermentation Quality, Bacterial Communities, and Ruminal Greenhouse Gas Emissions of Paper Mulberry Silage

**DOI:** 10.3389/fmicb.2021.820011

**Published:** 2022-02-11

**Authors:** Xiaomei Li, Fei Chen, Jingjing Xu, Linna Guo, Yi Xiong, Yanli Lin, Kuikui Ni, Fuyu Yang

**Affiliations:** ^1^College of Grassland Science and Technology, China Agricultural University, Beijing, China; ^2^Beijing Sure Academy of Biosciences, Beijing, China

**Keywords:** herbal residues, bacterial community, fermentation quality, greenhouse gases, paper mulberry, PacBio SMRT

## Abstract

This study aimed to investigate the influence of herbal residues on the fermentation quality and ruminal fermentation of paper mulberry silage. Clove, mint, and purple perilla residues were used as additives. Silage treatments were designed as control (no additives), 5% of clove, 5% of mint, and 5% of purple perilla. After 21 and 75 days of fermentation, the fermentation characteristics, bacterial communities, and ruminal greenhouse gas emissions *in vitro* incubation of paper mulberry were analyzed. The results showed that the used herbal residues could reduce the protein losses in paper mulberry silage based on the lower contents of ammoniacal nitrogen and nonprotein nitrogen. Compared with control, higher lactic acid and propionic acid contents were observed in the silages treated with mint and purple perilla but with a higher acetic acid content in clove treatment. Real-time sequencing technology (single-molecule real-time) revealed that *Lactobacillus* was the dominant bacteria in all silages at the genus level, whereas the bacterial abundance in the treated silages differed greatly from control at the species level. *Lactobacillus hammesii* abundance was the highest in control, whereas *Lactobacillus acetotolerans* was the first predominant in the treated silages. All the additives enhanced the digestibility of *in vitro* dry matter significantly. However, purple perilla decreased the production of total gas, methane, and carbon dioxide. The findings discussed earlier suggested that herbal residues have potential effects in improving fermentation quality, reducing protein loss, and modulating greenhouse gas emissions in the rumen of paper mulberry silage by shifting bacterial community composition.

## Introduction

In China, approximately 70 million tons of herbal residues are produced each year, but a large amount of them are discarded directly, which causes serious environmental pollution such as groundwater pollution, unpleasant smells, or greenhouse gases emissions (Zhang et al., 2017; Wang et al., 2021). However, herbal medicine residues still contain great nutrient content and natural bioactive bioactivity such as cellulose, proteins, flavonoids, polysaccharides, organic acids, and so on (Su et al., 2018; Ni et al., 2020). These biologically active substances exhibit antibacterial, antioxidant, and immune-enhancing properties and can be thought of as alternative, green additives that can replace antibiotics in promoting animal growth and improving the endurance and disease resistance of livestock (Falleh et al., 2020). Therefore, it is meaningful to search for an effective way to recycle this kind of biomass resource.

Ensiling is an important way for preserving forage and dealing with agricultural wastes. The fermentation quality mainly depends on the results of the competition between lactic acid bacteria and spoilage microorganisms, as well as the competition and collaboration among lactic acid bacteria species (Yan et al., 2019; Bai et al., 2021). In the past decades, many additives have been developed to improve ensiling quality by manipulating the microbial communities, such as inoculants (Xu et al., 2018), chemical fungicides (Ogunade et al., 2017), and plant secondary metabolites (Ni et al., 2020). Among them, plant secondary metabolites have aroused growing interest due to their desirable effects in inhibiting pathogenic microorganisms’ growth and enhancing animal performance (Niderkorn and Jayanegara, 2021). Especially, there were increasing concerns and challenges in using antibiotic or chemical fungicides in the livestock industry. More importantly, plant secondary metabolites could exert more consistent effects than microbial inoculants, as they are less dependent on biological processes.

Herbal residues are a kind of by-product produced after the extraction process from medicinal plant materials (Zhou et al., 2018; Wang et al., 2021). To date, there are approximately 1,600 kinds of Chinese medicinal herbs used in China (Zhou et al., 2018). Depending on the plant types, plant volatiles were found to have stimulatory, inhibitory, or no effect on microorganisms (Greff et al., 2020). Among them, aromatic herbal residues, such as clove, mint, and purple perilla, contain a variety of essential oils, phenolic compounds, flavonoids, and terpenoids. These biologically active substances exhibit highly stimulatory and/or inhibitory effects on a wide variety of bacteria and fungi (Falleh et al., 2020). In recent years, increasing secondary metabolite products were extracted from aromatic herbal plants and used in forage conservation. They not only inhibited deamination and proteolysis but also improved silage’s aerobic stability and *in vitro* digestibility (Cantoia et al., 2020; Çayıroğlu et al., 2020). However, little information was available on how herbal residues affect the microbial composition of silage.

It is well known that methane emission not only leads to global warming (Solomon et al., 2007) but also causes a great loss of feed energy based on the type of forage (Wang et al., 2016). Therefore, how to ameliorate rumen–methane emissions became an attractive study topic. Several types of phytochemicals, including saponins (Rira et al., 2015), tannins (Fitri et al., 2021), essential oils (Salman et al., 2018), and alkaloids (Rira et al., 2015), had positive actions on mitigating greenhouse gas emissions in the rumen through modulating proportions and concentrations of volatile fatty acids or suppressing methanogens. However, the successful suppression of methane was usually accompanied by a considerable reduction in feed digestion. Likewise, herbal residues with abundant phytochemicals might help reduce methane emissions. Therefore, it is significant to evaluate whether herbal residues in the ensiling environment could reduce greenhouse gas emissions from ruminants with no or little adverse effects on digestion.

Paper mulberry (*Broussonetia papyrifera* L.) is widely found in any country, such as Thailand, Africa, the United States, and China. It produces a large quantity of biomass each year, ranging from 45 to 120 tons per hectare (Zhang et al., 2019; Guo et al., 2021). Paper mulberry is rich in crude protein, amino acids, flavonoids, phenolic acids, and lignans, which have the potential to promote animal growth and disease resistance. These nutritional traits, along with high production and biologically active compounds, have turned paper mulberry into a sustainable, functional, and important protein forage resource for livestock (Cai et al., 2019; Xiong et al., 2021). However, the natural fermentation of paper mulberry is usually attributed to undesirable *Enterobacter* and *Clostridium* (Du et al., 2021; Guo et al., 2021), leading to extensive proteolysis and great economic losses. Based on the characteristics of herbal residues, we suspect that co-ensiling of herbal residues and paper mulberry might be a simple and efficient way to recycle herbal residues and produce good silage products.

In this study, three aromatic herbal residues, i.e., clove, mint, and purple perilla, were selected as the objects to investigate their effects on the fermentation quality, bacterial communities, and ruminal greenhouse gas emissions of paper mulberry silage. The results of this study might provide useful references for developing herbal residues as new additives for improving silage quality and sustainable mitigation of greenhouse gas emissions from ruminants.

## Materials and Methods

### Silage Preparation

Paper mulberry was manually collected from the experimental farm (108°63′N, 24°50′E) of China Agricultural University (Hechi, Guangxi, China) on November 8, 2020. The fresh paper mulberry was harvested at approximately 1.2 m high with 0.1 m stubble height, then directly crushed into small pieces (<1 cm) using a forage crusher (BH-188, Feilong Machinery Company, Guangxi, China). Three herbal plants residues were used as additives in this study, which are widely used in animal and human food. Clove (*Syzygium aromaticum* L.), mint (*Mentha canadensis* L.), and purple perilla (*Perilla frutescens* L.) after extracted through steam distillation for volatile oil were obtained from Beijing Tongrentang Co., Ltd., China. All the dry herbal residues were milled by passing through a 60-mesh screen using a multimill (KFJ-35, Runhao Machinery Manufacturing Co., Ltd., Zhejiang, China) and stored in the shade place. The silage treatments were as follows: (i) control, no additives, (ii) SA, 5% of clove, (iii) MC, 5% of mint, and (iv) PF, 5% of purple perilla. Then, 500-g crushed fresh paper mulberry or that added with 5% of herbal residues were mixed homogenously and packed manually into vacuum-sealed polyethylene plastic bags (dimensions 28 × 35 cm, Deli, Beijing, China; Guo et al., 2021). Six bags for each treatment were prepared and kept at room temperature (20–30°C). After 21 and 75 days of ensiling, three bags were randomly selected and opened to analyze the fermentation parameters, bacterial communities, and chemical composition (Guo et al., 2021).

### Fermentation and Chemical Profile Analysis

After 21 and 75 days of ensiling, the ensiling bags were opened. A portion of the silage sample was immediately frozen (−80°C) for bacteria community analysis. According to the report of Yan et al. (2019), with slight modifications, the populations of lactic acid bacteria, coliform bacteria, and fungi (molds and yeasts) were determined using de Man, Rogosa, and Sharpe agar, blue light agar (Nissui), and Rose Bengal agar, respectively. For fermentation parameters, 25-g samples were mixed with 225 ml of sterile water and stored at 4°C overnight and then filtered *via* four layers of cheesecloth (Li et al., 2021). Immediately after that, the pH value was measured, and a portion of filtrate was filtrated through 0.22-μm filters to determine the organic acid contents through high-performance liquid chromatography (KC-811, Shimadzu Co., Ltd., Kyoto, Japan). The ammonia-N was assessed as previously established (Guan et al., 2018).

A total of 27 fresh and silage paper mulberry samples were dried at 65°C for 3 days. All dried samples were milled by passing through a 40 mesh screen for chemical analysis. Total nitrogen content (TN) and crude protein (CP) were analyzed by the Kjeldahl method (AOAC, 1990). Nonprotein nitrogen (NPN) was assessed as described previously by Ke et al. (2017). The contents of acid detergent fiber (ADF) and neutral detergent fiber (NDF) were measured by Ankom 2000 fiber analyzer (Van Soest et al., 1991). Water-soluble carbohydrate (WSC) was estimated by the methodology of Thomas (2010).

### *In vitro* Rumen Incubation and Animal Care

Rumen fluid was collected before morning feeding on three adult small-tail Han sheep (corn–soybean meal diet with alfalfa hay). The rumen fluid was kept in warm insulated flasks that had been prewarmed and filled with CO_2_ to ensure an anaerobic environment. The silage samples (approximately 0.2 g of DM) were weighed in 120-ml glass bottles with 10 repeats in advance, and three blank controls were also prepared. Incubation fluid (30 ml), made of 10 ml of rumen fluid that had filtered through four layers of cheesecloth and 20 ml of buffer solution, was added to each bottle, and then all the bottles were incubated at 39°C (Menke and Steingass, 1988; Guo et al., 2019; Fant et al., 2020). The cumulative total gas production was recorded at 0-, 2-, 4-, 6-, 8-, 12-, 24-, 36-, and 48-h incubations. After 48 h of incubation, the gas of each bottle was collected using aluminum foil bags (HBI, Shanghai Bestest Biological Technology Co., Ltd., Shanghai, China). Gas composition was analyzed in accordance with the methods reported by Wang et al. (2019). The fermentation slurry of each bottle was collected and immediately centrifuged for 10 min at 3,000 × *g* at 4°C. The supernatant was used to determine the pH level and VFA levels as aforementioned.

*In vitro* dry matter digestibility (IVDMD), *in vitro* neutral detergent fiber digestibility (IVNDFD), and *in vitro* acid detergent fiber digestibility (IVADFD) were determined with an Ankom DaisyII incubator (Ankom Technologies, Macedon, NY, United States). The experiment was conducted in accordance with the Chinese Guidelines for Animal Welfare and Experimental Protocol and approved by the Animal Care and Use Committee of China Agricultural University.

### Bacterial Community Analysis

The method of DNA extraction is according to Guan et al. (2018). The quality and concentration of the extracted DNA were monitored as described specifically by Yan et al. (2019). The polymerase chain reaction amplification of the full-length 16S ribosomal RNA gene was performed with the primer 27F (5′-GAGAGTTTGATCCTGGCTCAG-3′) and the reverse primer 1492R (5′-TACCTTGTTACGACTT-3′), and the polymerase chain reaction program was as follows: 95°C for 2 min; 25 cycles of 98°C for 10 s, 55°C for 30 s, and 72°C for 90 s, with a final extension of 72°C for 2 min. Sequence preprocessing was carried out on a PacBio RS II instrument using P6-C4 chemistry (Yan et al., 2019).

Raw circular consensus sequencing reads were obtained with Single-Molecule Real-Time Portal version 2.7 (PacBio). Then, the sequence extraction, filtering, and optimization were executed as described by Li et al. (2021). The unique sequence set was classified into operational taxonomic units based on a 97% threshold identity using UCLUST (Kim et al., 2014). Subsequently, the representative sequence was compared using the Mothur3 software with the Silva database to gain classified information (Li et al., 2021). Alpha diversity indices were calculated using QIIME software. The principal component analysis was conducted to assess the structural variation of microbiota. Heatmap analysis was performed using an R-based statistics tool to identify the correlation between fermentation characteristics and relative abundance of the silage bacteria species. Linear discriminant analysis effect size (LEfSe) analyses were conducted using a free online platform (Guo et al., 2021).

### Statistical Analysis

Analysis of variance (ANOVA) was conducted *via* generalized linear modeling in SPSS to determine the significant difference among the samples. The significance was set at *P* < 0.05. The data from chemical composition, fermentation characteristics, microbial population, and alpha diversity of the silages were subjected to two-way ANOVA with a fully randomized design, with ensiling time (D) and additives (M) as the main variables. The data concerning the cumulations of total gas, CH_4_ production, CO_2_ production, and *in vitro* digestibility were subjected to one-way ANOVA. Mean values were compared using Tukey’s test.

## Results and Discussion

### Chemical Characteristics of Raw Materials

The chemical characteristics of the raw materials before ensiling are listed in Table 1. The dry matter (DM) of paper mulberry was 324.45 g/kg, and its CP, NDF, and ADF contents were 146.12, 440.03, and 269.33 g/kg DM, respectively. Different from previous reports (Du et al., 2021; Guo et al., 2021), higher fiber and lower CP contents of paper mulberry in our study might be related to the growth period, harvest time, and cold climatic conditions. In late fall and winter, plants translocate nitrogen from the aboveground parts to the roots to produce new growth the following spring (Waramit et al., 2012; Liu et al., 2016). WSC was used as the major substrates for microbial growth, especially LAB, during the ensiling process. In this study, the WSC content was 87.37 g/kg, which was beyond the minimal requirement for good silage quality (Mcdonald et al., 1991). Besides, the CP content of the herbal residues used ranged from 65.33 to 226.55 g/kg DM, and the highest CP content was found in PF. Meanwhile, the WSC contents in SA and PF exceeded 138 g/kg DM but with lower NDF and ADF contents.

**TABLE 1:** Chemical characteristics of raw materials and silages after 21 and 75 days of ensiling.

Sample ID		DM (g/kg)	CP (g/kg DM)	NPN (g/kg TN)	NDF (g/kg DM)	ADF (g/kg DM)	HC (g/kg DM)	WSC (g/kg DM)
**Raw material**								
Paper mulberry		324.45 ± 3.09	146.12 ± 2.11	-	440.03 ± 2.06	269.33 ± 1.00	170.70 ± 3.03	87.37 ± 0.82
Mint		872.73 ± 2.73	88.39 ± 1.41	-	445.09 ± 1.81	337.15 ± 1.17	107.90 ± 2.99	49.24 ± 1.76
Clove		870.39 ± 1.08	65.33 ± 0.42	-	238.14 ± 5.88	179.99 ± 2.71	58.15 ± 3.18	144.35 ± 0.29
Purple perilla		871.21 ± 2.39	226.55 ± 0.70	-	300.97 ± 4.91	213.85 ± 3.88	87.12 ± 2.71	138.29 ± 0.76
**Silage samples**								
CK	21 D	333.32 b	134.09 c	402.8 b	433.42 a	261.34 a	172.09 ab	13.07 de
	75 D	335.99 b	142.70 b	429.99 a	418.82 b	254.54 ab	156.78 d	11.73 e
MC	21 D	351.06 a	131.26 cd	378.37 cd	406.97 c	254.69 ab	152.28 d	14.59 bc
	75 D	356.81 a	137.34 bc	391.75 bc	406.03 c	236.3 de	169.74 abc	13.95 cd
SA	21 D	354.96 a	126.35 d	353.46 e	403.1 c	244.17 bc	158.93 cd	15.45 ab
	75 D	357.57 a	132.28 cd	344.15 e	404.09 c	241.15 cd	162.94 bcd	16.29 a
PF	21 D	355.82 a	150.85 a	373.36 d	415.73 bc	245.76 bc	169.96 abc	13.64 cd
	75 D	358.42 a	154.20 a	387.87 bc	411.69 bc	229.63 e	182.06 a	12.71 de
SEM		0.20	1.91	5.31	2.10	2.13	1.99	0.32
*P*-value	D	0.023	< 0.001	<0.001	0.036	< 0.001	0.022	0.150
	M	< 0.001	<0.001	< 0.001	<0.001	< 0.001	<0.001	< 0.001
	D × M	0.808	0.266	0.001	0.057	0.022	< 0.001	0.164

*^a–e^Means of additives treatments within a column with different superscripts differ (P < 0.05). CK, without additives; MC, added 5% mint; SA, 5% clove; PF, 5% purple perilla; DM, dry matter; CP, crude protein; NPN, nonprotein nitrogen; NDF, neutral detergent fiber; ADF, acid detergent fiber; HC, hemicellulose; WSC, water-soluble carbohydrate; D, ensiling days; M, herbal residues; D × M, interaction of ensiling days and treatment; SEM, standard error of means.*

### Fermentation Characteristics and Chemical Composition of Ensiling

The fermentation characteristics of paper mulberry silages during ensiling are presented in Table 2. Overall, herbal residues and the interaction of ensiling time × herbal residues had significant (*P* < 0.01) effects on pH, lactic acid, acetic acid, propionic acids, and the ratio of lactic to acetic acid compared with control. The pH value is always considered an important indicator for reflecting microbial activity in the process of ensiling. The ensiling aimed to reduce the pH of the silage as rapidly as possible to ≤ 4.2 and preferably to ≤ 4.0 (Mcdonald et al., 1991). In our study, all silage samples showed a low pH value (≤ 4.2) at the early stage of ensiling, especially in silages treated with mint (*P* < 0.05). However, our results differed from previous studies, which showed the pH value of paper mulberry silage at 4.7–5.7 after 60 days of fermentation (Zhang et al., 2019; Guo et al., 2021). The difference discussed earlier could be attributed to the high WSC content in our study, which could provide more substrates for further pH decline.

**TABLE 2:** Fermentation characteristics of paper mulberry silage after 21 and 75 days of ensiling.

Item	Ensiling days	CK	MC	SA	PF	SEM	*P*-value		
							D	M	D × M
**Fermentation quality**									
pH	21	4.14 b	4.10 c	4.15 b	4.20 a	0.01	< 0.001	<0.001	0.001
	75	4.10 b	4.04 c	4.09 b	4.22 a	0.02			
NH3-N/TN	21	4.61 a	3.76 c	3.28 d	4.08 b	0.14	< 0.001	<0.001	0.002
	75	5.61 a	5.02 b	3.59 c	4.70 b	0.22			
Lactic acid (g/kg DM)	21	40.81 c	47.41 a	32.54 d	44.17 b	1.57	< 0.001	< 0.001	< 0.001
	75	65.05 c	82.50 a	55.02 d	76.02 b	3.03			
Acetic acid(g/kg DM)	21	10.10 c	12.45 b	15.28 a	11.58 bc	0.58	< 0.001	<0.001	0.018
	75	18.66 c	23.47 b	28.03 a	19.86 c	1.11			
Propionic acid (g/kg DM)	21	1.66 b	10.98 a	3.42 b	14.03 a	1.51	< 0.001	<0.001	0.028
	75	13.27 b	22.91 a	15.30 b	16.44 ab	1.41			
Butyric acid (g/kg DM)	21	nd	nd	nd	nd	-	-	-	-
	75	nd	nd	nd	nd	-			
Lactic acid/Acetic acid	21	4.04 a	3.81 a	2.13 b	3.81 a	0.22	0.278	< 0.001	0.009
	75	3.49 a	3.52 a	1.96 b	3.83 a	0.24			
**Microbial population (log10 cfu/g of FM)**									
Lactic acid bacteria	21	7.10 a	6.94 a	5.74 b	7.02 a	0.16	< 0.001	<0.001	0.188
	75	5.93 a	5.81 a	4.86 b	5.92 a	0.13			
Coliform bacteria	21	nd	nd	nd	nd	-	-	-	-
	75	nd	nd	nd	nd	-			
Yeast	21	nd	nd	nd	nd	-	-	-	-
	75	nd	nd	nd	nd	-			

*^a–d^Means of additives treatments within a row with different superscripts differ (P < 0.05). CK, without additives; MC, 5% mint; SA, 5% clove; PF, 5% purple perilla; SEM, standard error of means; DM, dry matter; NH_3_-N/TN, ratio of ammonia nitrogen/total nitrogen; FM, fresh matter; cfu, colony-forming unit; nd, not detected; D, ensiling days; M, herbal residues; D × M, interaction of ensiling days and treatment.*

As shown in Table 2, lactic acid, acetic acid, and propionic acid were mainly added in all silages, but their amounts differed greatly in various treatments. The addition of mint and purple perilla significantly increased (*P* < 0.05) the lactic acid content, especially in silages treated with mint. In contrast, the clove-treated silage had the lowest lactic acid content in all treatments. Besides, we also found that silages treated with clove had lower counts of culturable LAB. The findings mentioned earlier were consistent with the previous data, in which LAB strains could not be inhibited by the *Mentha* or its extraction (Fancello et al., 2017). However, SA was found to have a high antimicrobial activity on LAB and other microorganisms because the components of clove, such as eugenol, β-caryophyllene, and α-humulene, can induce cell wall degradation, disrupt the cytoplasmic membrane, and then accelerate cell death (Kang et al., 2019; Takahashi et al., 2021).

The ratio of lactic acid to acetic acid was commonly used as a qualitative indicator of fermentation. In the present study, the ratio of lactic acid to acetic acid in control, PF, and MC treatments were higher than 3.0. Interestingly, clove-treated silages induced higher acetic acid and lower lactic acid contents than other silages, leading to a decrement in the ratio of lactic acid to acetic acid, especially after 75 days of ensiling (Table 2). That difference might result from the variable susceptibility of LAB to herbal plants or their components. Fancello et al. (2016) reported that *Lactobacillus acidophilus*, *Lactobacillus plantarum*, and *Lactobacillus rhamnosus* had a high sensitivity to the components of citrus lemon leaves, whereas *Lactobacillus paracasei* showed a lower sensitivity. In addition, *Pediococcus acidilactici* was the most resistant LAB to the essential oils of clove bud, cinnamon bark, and thyme, whereas *Lactobacillus buchneri* was moderately tolerant, and *Leuconostoc citrovorum* was one of the most susceptible LAB plants (Takahashi et al., 2021). Therefore, we supposed that the clove had a potential effect in improving the growth of heterofermentative lactic acid bacteria and metabolism and led to the fermentation pattern toward acetate in the paper mulberry silage. The butyrate was not detected in all silages. That might be attributed to the rapid drop of pH, and *Clostridia* was unable to ferment sugars to butyric acid or convert lactic to butyric acid (Kung et al., 2018).

The primary goal of ensiling was to maximize the preservation of nutrients, especially CP, as shown in Tables 1, 2. The factorial analysis revealed that herbal residues had significant (*P* < 0.01) effects on crude protein, NH_3_-N/TN, NPN, fiber, and WSC. In addition, the interaction of ensiling time × herbal residues significantly influenced NH_3_-N/TN, NPN, and hemicellulose. Lower NH_3_-N/TN and NPN contents were observed compared with those in previous studies (Zhang et al., 2019; Guo et al., 2021), which reported the content of NH_3_-N/TN among 60–120 g/kg TN. According to Lindgren et al. (1983), proteolysis was high during the early stage of ensiling and decreased as pH reduced in silages. The highest proteolysis occurred at pH between 5.5 and 6.0 and was reduced by 65–85% at pH 4.0. In the present study, a higher acidification rate might make epiphytic LAB higher than proteolysis microorganisms, such as enterobacteria or clostridia, because LAB was more resistant to hyper-acidification because they could withstand and survive in a relatively low pH environment (Kung et al., 2018) and avoid excessive protein degradation. Although all silages had a lower pH value after 21 days of ensiling, we need to highlight that the medicinal and edible homologous plants used reduced NH_3_-N/TN and NPN contents, wherein the lowest NH_3_-N and NPN contents were found in SA treatment. Strong antimicrobial activity of the additives used was one of the possible mechanisms for the preservation of CP in the present study. Herbal plants or their components have positive effects in inhibiting ammonia-producing bacteria (Mcintosh et al., 2003), which was even more significant in an acidified environment (Falleh et al., 2020). The bacteria susceptibility to extracts from herbal plants increased at a low-pH environment, as the extracts were more hydrophobic at this condition, with an increased dissolving ability in the bacteria membrane (Falleh et al., 2020). On the other hand, herbal plants or their components damaged the integrity of cell membranes, thus increasing the vulnerability of bacteria to acidic environments caused by organic acids (Karatzas et al., 2001). Although the DM loss did not show significant differences in all silages, silages treated with herbal plants residues had higher hemicellulose and lower ADF and NDF contents compared with control as the ensiling duration prolonged (Table 1). The results suggested that the application of herbal residues could improve the fiber structure of silage quality.

As the dominant bacteria during ensiling, LAB contained a wide array of bacterial species with different phylogenetic and fundamental characteristics. As mentioned previously, herbal plants or their components exerted various effects on LAB species, and LAB species showed different sensitivity to the components of the same herbal plants. Therefore, an in-depth understanding of microbial communities and their relative abundances in silages treated with herbal plants would facilitate improving the preservation of silage and the recycling use of herbal plants efficiently as functional and healthful silage additives.

### Dynamic Changes of Bacterial Communities

The bacterial diversity of paper mulberry silages is shown in Table 3. The coverage value of all samples was more than 0.99, indicating that the sequencing depth was sufficient for understanding the microbial composition. Lower values of operational taxonomic units, Chao1, and abundance-based coverage estimator were observed in the treated silages after 75 days of ensiling compared with control, which indicated that the substantial selection of bacteria occurred after herbal residues were added.

**TABLE 3:** Alpha-diversity of bacterial diversity of paper mulberry silage.

Treatments		Operational taxonomic unit	Abundance-based coverage estimator	Chao1	Simpson	Shannon	Coverage
CK	21 d	72	97.79	107.39	0.70	2.27	0.997
	75 d	145	167.66	164.91	0.85	3.40	0.997
MC	21 d	109	176.55	166.05	0.44	1.67	0.994
	75 d	72	165.15	109.54	0.23	0.87	0.997
SA	21 d	79	146.05	117.39	0.76	2.50	0.996
	75 d	122	150.50	149.40	0.75	2.90	0.996
PF	21 d	113	150.63	142.74	0.55	2.12	0.997
	75 d	99	134.39	139.60	0.62	2.08	0.997

*CK, without additives; MC, 5% mint; SA, 5% clove; PF, 5% purple perilla.*

**FIGURE 1:**
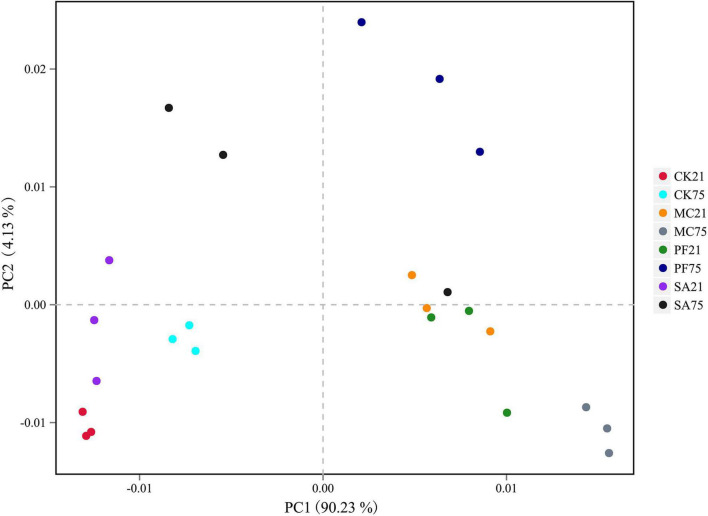
Principal component analysis (principal component) based on OTU level of paper mulberry silage. CK21, without additives after 21 days of ensiling; CK75, without additives after 75 days of ensiling; MC21, 5% mint after 21 days of ensiling; MC75, 5% mint after 75 days of ensiling; SA21, 5% clove after 21 days of ensiling; SA75, 5% clove after 75 days of ensiling; PF21, 5% purple perilla after 21 days of ensiling; PF75, 5% purple perilla after 75 days of ensiling.

The principal component analysis clearly reflected the variance of the bacterial communities of the silages (Figure 1). After 21 days of ensiling, the bacterial communities in control and SA treatment were clearly separated from the MC and PF groups, which suggested that the bacterial communities changed remarkably after the addition of mint and purple perilla at the early stage of ensiling. However, it was noteworthy that the bacterial communities in the four groups were separated clearly from each other as the ensiling time prolonged. In addition, the silages stored at the two time points were also separated from each other. It indicated that both herbal residues and ensiling time had an effect on the bacteria communities and fermentation quality of paper mulberry silage. Previous studies reported that the sensitivity to herbal plants or their components varied among bacteria species (Viuda-Martos et al., 2008; Takahashi et al., 2021), as the bioactive compounds exerted their antimicrobial activity through different mechanisms. Besides, as the ensiling time prolonged, fermentation might cause decomposition and/or biotransformation components of herbal plants ingredients, thereby modulating the product properties or changing the quantity of certain bioactive compounds (Hussain et al., 2016). Especially, the stable low-acidic environment enhanced the antibacterial activity of phenols or terpenes (Karatzas et al., 2001; Reddy et al., 2020) and then modified the bacterial communities of paper mulberry silage.

**FIGURE 2:**
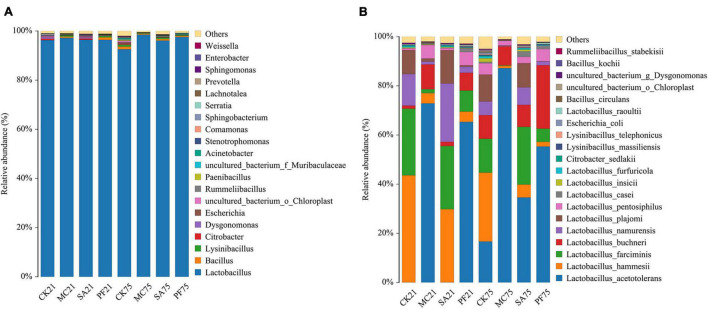
Relative abundances of silage bacterial composition at genus level **(A)** and species levels **(B)** after 21 and 75 days of ensiling.

Generally, successful fermentation paper mulberry silage, as well as forage fermentations, required faster growth of LAB than other undesirable microorganisms, such as *Enterobacterium* and *Clostridium*, so that LAB can quickly dominate the silage fermentation system before other microorganisms and have the chance to increase substantially in numbers and exert their effects. As shown in Figure 2, most of the undesirable microorganisms were quickly inhibited at the anaerobic condition at the early stage of ensiling, which was confirmed in the present study because *Lactobacillus* occupied over 95% in all silages (Figure 2A). As reported, the relative abundance of *Lactobacillus* ranged from approximately 20 to 70%, and *Enterobacter* also had a high relative abundance ranging from approximately 20 to 50% in the naturally fermented paper mulberry silages (Zhang et al., 2019; Du et al., 2021; Guo et al., 2021). This discrepancy might be attributed to the fast acidifying environment in the present study because *Enterobacter* was intolerant to the low pH (Queiroz et al., 2018). Besides, after long-term ensiling, the addition of herbal plants slightly increased or retained the relative abundance of *Lactobacillus*, whereas it showed a decreasing trend in control silages. In turn, the relative abundances of *Bacillus* and *Escherichia* were increased in control silages.

Although all samples had a high relative abundance of *Lactobacillus* (Figure 2A), different LAB species were observed in the four groups during ensiling (Figure 2B), which might have different functions because of their different metabolites. After 21 days of ensiling, the *Lactobacillus* species in control mainly contained *L. hammesii* (43.54 %), *Lactobacillus farciminis* (27.09 %), *Lactobacillus namurensis* (12.85 %), and *Lactobacillus plajomi* (9.80%). Silages treated with clove had similar bacterial species with control but differed in their relative abundance. However, higher relative abundances of *Lactobacillus acetotolerans* (65.31–72.83%) and *L. buchneri* (7.24–10.06%) were observed in MC and PF treatments. The results indicated that mint and purple perilla residues might have a potential effect in increasing the abundance of *L. acetotolerans* at the early stage of ensiling. As the ensilage time prolonged, dominant *Lactobacillus* species in control became complex, such as *L. acetotolerans*, *L. hammesii, L. farciminis*, *L. plajomi, L. namurensis*, and *L. buchneri* (relative abundance > 5%). Interestingly, mint and purple perilla simplified the bacterial structure and changed the *Lactobacillus* species to *L. acetotolerans*, especially in silages added with mint whose relative abundance of *L. acetotolerans* was as high as 87.19%.

**FIGURE 3:**
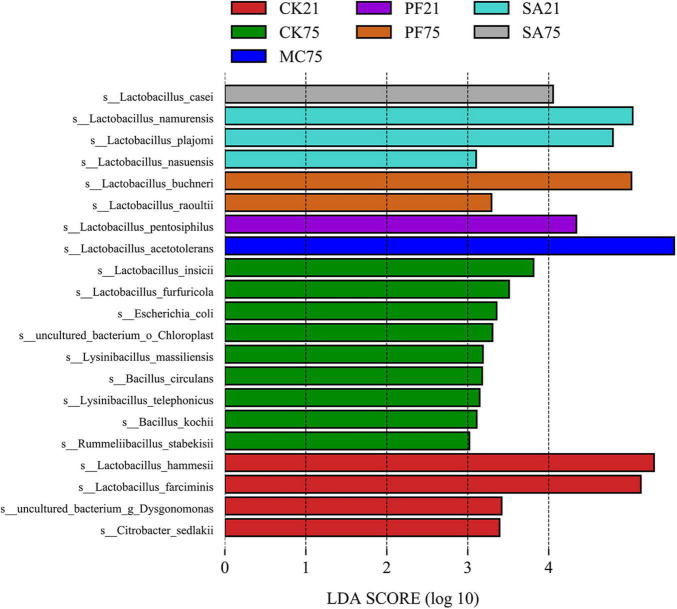
Linear discriminant analysis effect size analysis of bacterial community (LDA score > 3.0) at species level in paper mulberry silages prepared by different herbal residues.

*L. acetotolerans*, the most dominant species found in MC and PF treatments, was also identified as the most representative bacterium in silages treated with mint by the LEfSe algorithm (Figure 3). That indicated that *L. acetotolerans* with a better tolerance on mint and purple perilla was more competitive than other LAB species in our study. As a kind of facultative heterofermentative LAB, *L. acetotolerans* could produce lactic acid and acetic acid from sugars (Entani et al., 1986). In particular, *L. acetotolerans* was highly acid-tolerant during the fermentation period, with different and some physiological properties and metabolic functions from other *Lactobacillus* species (Entani et al., 1986; Xu et al., 2020); for instance, *L. acetotolerans* was positively correlated with 3-phenyllactic acid and azelaic acid (Xu et al., 2018), which might be an important contributory factor to the less bacterial diversity in MC and PF treatments after 75 days of ensiling (Figure 2). Besides, Han et al. (2018) reported that *L. acetotolerans* in the silage was detected in the cow gut based on qualitative DGGE assessment. Therefore, mint and purple perilla-added silages with a high relative abundance of *L. acetotolerans* could be regarded as an important indicator for the propagation and delivery of probiotics in animals. *L. hammesii*, *L. buchneri*, and *L. namurensis* were heterofermentative, whereas *L. farciminis* was homofermentative. All of them were used as silage inoculants to improve preservation efficiency. The high abundance of heterofermentative LAB, such as *L. hammesii* and *L. namurensis*, in SA treatment contributed to the higher acetic acid content. Besides, based on LEfSe analysis (Figure 3), 13 species-level bacterial taxa were found to be differentially abundant in control silage compared with the treated silages, such as *Escherichia coli*, *Bacillus circulans*, *Lysinibacillus massiliensis*, and *Citrobacter sedlakii*. Most of them were undesirable and could cause the decomposition of sugars and proteins in forage (Queiroz et al., 2018; Guo et al., 2021), which contributed to the lower fermentation quality and higher loss of NPN in control silages.

### Correlation Analysis of Bacterial Communities With Fermentation Characteristics

**FIGURE 4:**
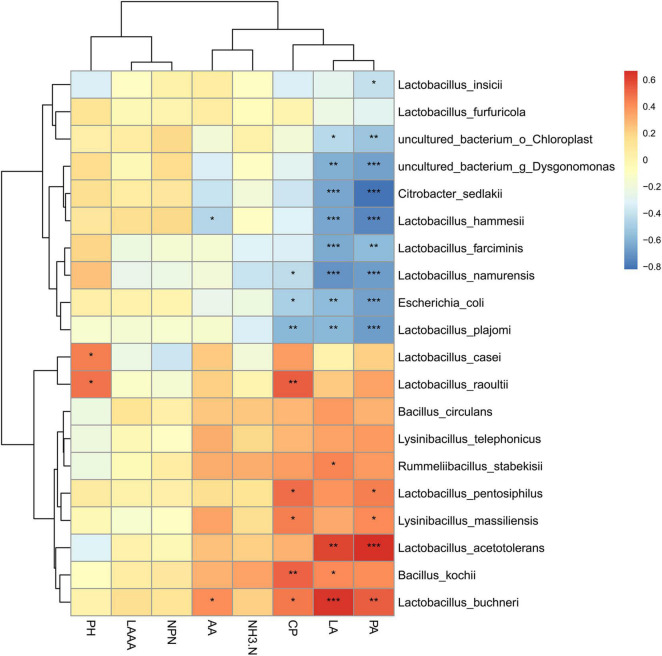
Correlation analysis between bacterial community and fermentation products in paper mulberry silage. Corresponding value of middle heat map is Spearman correlation coefficient r, which ranges between 1 and 1, *r* < 0 indicates a negative correlation (blue), *r* > 0 indicates a positive correlation (red), and *, **, and *** represent *P* < 0.05, *P* < 0.01, and *P* < 0.001, respectively.

To further evaluate the potential correlation between bacterial communities and fermentation performance of paper mulberry silage treated with or without herbal residues, the relationships between bacteria species were evaluated based on the variation in pH, organic acid content, and NH_3_-N/TN (Figure 4). As expected, *L. acetotolerans* was positively correlated with the lactic acid and propionic acid contents; and *E. coli* was negatively correlated with the concentrations of CP. It was worth noting that *L. hammesii* was negatively correlated with the acetic acid content, whereas *L. buchneri* was positively correlated with the lactic acid content; both of them were heterofermentative bacteria, illustrating that some of *Lactobacillus* species might not directly affect the fermentation characteristics as the ensiling period prolonged. *Bacillus kochii* was detected at a low level in paper mulberry silage, although it showed a positive correlation with CP.

### *In vitro* Digestibility and Gas Production of Paper Mulberry Silage

As shown in Table 4, herbal residues had significant (*P* < 0.05) effects on IVDMD, IVNDFD, and IVADFD. All treated silages had higher IVDMD compared with control. Besides, the application of clove and purple perilla significantly increased the IVNDFD. As reported, high fiber feed could result in more particulate DM in feces and a larger mean particle size but a decreased digestibility (Schulze et al., 2014; Hao et al., 2021). In addition, the bioactive components of herbal plants also had the potential effect in promoting *Fibrobacter succinogenes*, *Ruminococcus flavefaciens*, fungi, and so on in rumen, thereby improving ruminal degradability (Kim et al., 2019). Therefore, the greater fermentation quality and the potential bioactive substance in treated silage seemed correlated with the higher paper mulberry silage digestibility in this study.

**TABLE 4:** *In vitro* digestibility and rumen fluid fermentation profile of paper mulberry silage after 75 days of ensiling.

Item	CK	MC	SA	PF	SEM	*P*-value
pH	7.01	7.11	6.91	7.08	0.23	0.078
Acetic acid (mmol/L)	51.48ab	49.24b	52.32a	45.15c	0.88	0.002
Propionic acid (mmol/L)	21.67b	23.97a	21.10b	23.49a	0.57	0.015
Butyric acid (mmol/L)	14.46b	15.79a	15.03ab	14.18b	0.23	0.052
Acetic acid/propionic acid	2.38ab	2.05bc	2.48a	1.92c	0.09	0.017
IVDMD (g/kg DM)	671.16b	708.98a	716.41a	729.04a	6.69	0.001
IVNDFD (g/kg DM)	519.95b	518.31b	564.29a	593.56a	12.31	0.015
IVADFD (g/kg DM)	480.36ab	446.28a	462.28a	503.18a	10.91	0.035

*^a–c^Means of additives treatments within a row with different superscripts differ (P < 0.05). DM, dry matter; IVDMD, in vitro dry matter digestibility; IVNDFD, in vitro neutral detergent fiber digestibility; IVADFD, in vitro acid detergent fiber digestibility; CK, without additives; MC, 5% mint; SA, 5% clove; PF, 5% purple perilla; SEM, standard error of means.*

With regard to fermentation parameters, acetic acid, propionic acid, and butyric acid were the predominant VFAs produced by ruminal carbohydrate fermentation. Herbal residues significantly (*P* < 0.05) influenced acetic acid, propionic acid, and acetic acid/propionic acid. As shown in Table 4, the higher acetic acid and lower propionic acid were observed in SA treatment during *in vitro* incubation compared with control silage. On the contrary, both mint and purple perilla-treated silages induced the higher propionic acid and lower acetic acid in rumen fluid later, indicating that the application of mint and purple perilla could inhibit the acetate fermentation with the promotion of propionate fermentation, consequently leading to a decrement in the ratio of acetic acid to propionic acid, as well as a more desired change in the fermentation pattern without any drastic effects on ruminal pH. Pattanaik et al. (2018) recently reported that the differences in species and doses of plant additives contributed to the VFA variations during *in vitro* incubation, which could explain the observed variations among additives in the present study. Besides, it should be noted that the decreased ratio of acetic acid to propionic acid in PF treatment was inconsistent with Wang et al. (2016), who reported that the extracts of *P. frutescens* had no adverse effect on VFAs and the ratio of acetic acid to propionic acid of feed during *in vitro* incubation. This difference might be related to the changes in bioactive components of *P. frutescens* during ensiling. The fermentation process could improve the biological properties of plants *via* decomposition and/or biotransformation of complex substrates, which had a positive effect in modulating microbes and their metabolisms (Hussain et al., 2016).

**FIGURE 5:**
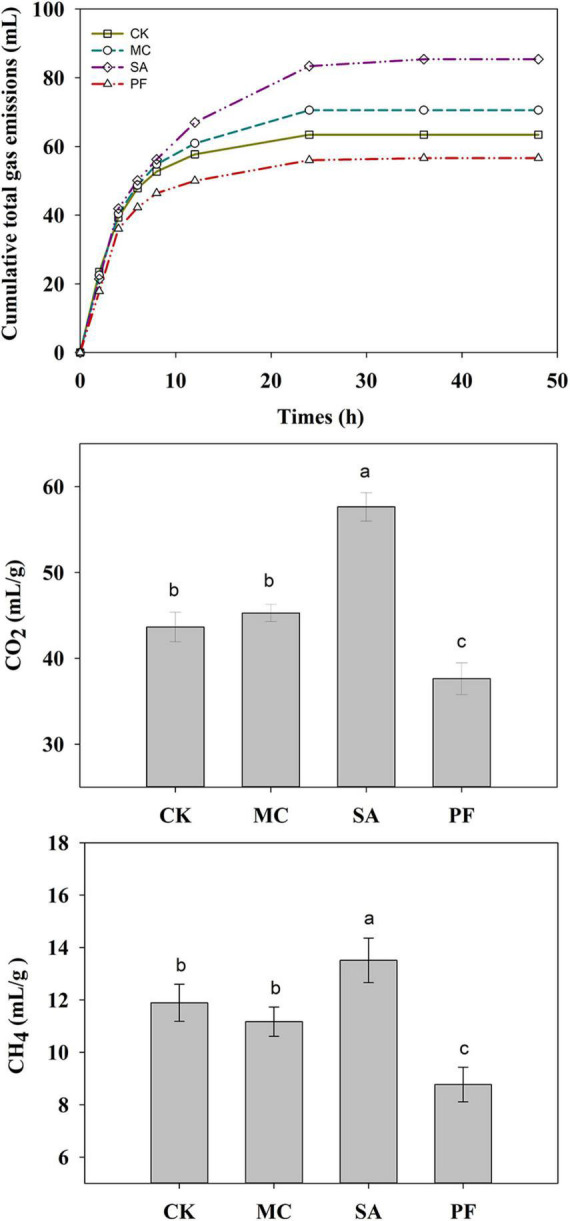
Cumulative total gas emissions, CH_4_, and CO_2_ production of paper mulberry silage after 75 days of ensiling during *in vitro* incubation. CK, without additives; MC, 5% mint; SA, 5% clove; PF, 5% purple perilla. ^a –c^Means with different superscripts are significantly different (*P* < 0.05).

**FIGURE 6:**
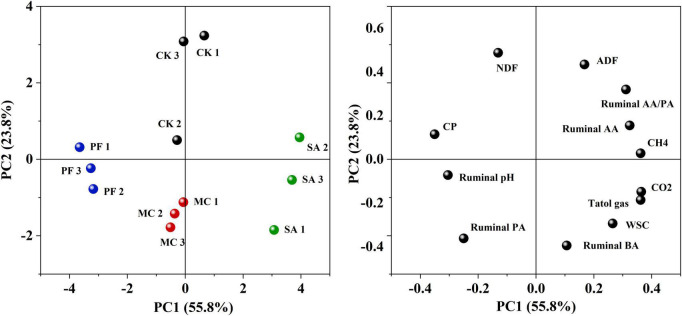
PCA biplots of separation of treatments and distribution of all variables after 75 days of ensiling. CK, without additives; MC, 5% mint; SA, 5% clove; PF, 5% purple perilla. CP, crude protein; NDF, neutral detergent fiber; ADF, acid detergent fiber; WSC, water-soluble carbohydrate; AA, acetic acid; PA, propionic acid; BA, butyric acid.

The gas from rumen fermentation contained mainly CH_4_ and CO_2_. In the present study, approximately 79–91% of the cumulative total gas was produced within 12 h of incubation (Figure 5). The application of clove and mint increased the production of cumulative total gas. Clove-treated silage increased the cumulation of CH_4_ and CO_2_ production compared with control, whereas MC treatment had a lower CH_4_ and higher CO_2_ production. In general, rumen gas is mainly produced by microbial consumption of saccharides and other nutrients. The high cumulative total gas in SA and MC treatment might be attributed to higher silage quality, especially in SA treatment, in which the WSC content was positively correlated to total gas, and fiber content was negatively correlated to total gas (Figure 6). However, the cumulative total gas productions and CH_4_ and CO_2_ production were the lowest in PF treatment, but with a higher IVDMD than other treated silages, indicating that co-ensiling paper mulberry with purple perilla residues might be a good way to inhibit greenhouse gas emissions with no adverse effects on digestion. One possible reason for these variations of gas was the change of ruminal fermentation pattern, in which AA/PA ratio is positively correlated to CH_4_ production and propionic acid is negatively correlated to CH_4_ production (Figure 6). In this study, purple perilla promoted propionate fermentation, and the higher production of propionate could compete for hydrogen with rumen methanogens. Therefore, the lower ruminal CH_4_ in PF treatment might be due to the redirecting hydrogen flow to other electron acceptors, including propionate (Mcallister and Newbold, 2008; Zhao et al., 2019).

## Conclusion

The herbal residues used greatly improved fermentation parameters and *in vitro* digestibility of silages, as evidenced by the lower NH_3_-N, loss of crude protein, and higher *in vitro* dry matter and fiber digestibility of paper mulberry. In addition, they clearly modulated the bacterial communities of paper mulberry silage during ensiling, especially in *Lactobacillus* species. Besides, the inclusion of purple perilla residues could reduce rumen CH_4_ and total greenhouse gas emissions of paper mulberry silage. This study provided new insight into the sustainable improvement of silage fermentation, which will enhance the future development of more functional and healthful silage additives and sustainable mitigation of greenhouse gas emissions from ruminants.

## Data Availability Statement

The raw data supporting the conclusions of this article will be made available by the authors, without undue reservation.

## Ethics Statement

The animal study was reviewed and approved by Animal Care and Use Committee of China Agricultural University.

## Author Contributions

XL, KN, and FY designed the study and wrote the manuscript. FC, JX, and LG performed the experiments. YX and YL conducted the statistical and bioinformatics analysis. KN and FY were involved in the revision of the manuscript. All the authors reviewed and approved the final manuscript.

## Conflict of Interest

The authors declare that the research was conducted in the absence of any commercial or financial relationships that could be construed as a potential conflict of interest.

## Publisher’s Note

All claims expressed in this article are solely those of the authors and do not necessarily represent those of their affiliated organizations, or those of the publisher, the editors and the reviewers. Any product that may be evaluated in this article, or claim that may be made by its manufacturer, is not guaranteed or endorsed by the publisher.
